# Traumatic Rupture of the Patellar Tendon From the Tibial Tuberosity in an Adult: A Case Report

**DOI:** 10.7759/cureus.19050

**Published:** 2021-10-26

**Authors:** Saurabh Pagdal

**Affiliations:** 1 Arthroscopic Surgery, Dr Pagdal Ortho Clinic, Sangamner, IND

**Keywords:** patellar tendon rupture, semitendinosus and gracilis graft, ligament reconstruction, ligament repair, trauma, knee, tendon, injuries, patellar tendon avulsion

## Abstract

Patellar tendon ruptures from the tibial tuberosity are very uncommon. Various surgical techniques are described for patellar tendon ruptures from the tibial tuberosity. A 58-year-old male without any predisposing factors had pain and swelling in the right knee due to a road traffic accident. Tense swelling and ecchymosis were present around the right knee with a palpable defect over the patellar tendon and an inability to extend the right knee. An MRI report revealed avulsion of the distal patellar tendon from the tibial tubercle insertion and medial meniscus tear. Primary repair was done with a double-loaded suture anchor and augmentation was done by using a Gracilis tendon. After the months, the knee range of motion (ROM) of the patient reached up to 90 degrees, and there was no extension lag on straight leg raise. Hamstring augmentation with a primary repair is the safest and a good surgical option in elderly patients with patellar tendon ruptures from the tibial tuberosity.

## Introduction

The incidence of patellar tendon rupture is less than that of rupture of the quadriceps tendon [1-2]. Furthermore, patellar tendon ruptures from the tibial tuberosity are very uncommon [3-4]. An acute traumatic patellar tendon rupture in adults is common in the third and fourth decades of life [1,3,5]. But in our case, an acute distal patellar tendon rupture occurred in a 58-year-old male due to a road traffic accident.

Various surgical techniques are described for patellar tendon ruptures from the tibial tuberosity, including end-to-end suture repair with stainless steel wires cerclage reinforcement [6], primary repair and allograft augmentation [7], primary repair with suture anchor [3], and SpeedBridge repair [8]. But challenges are plenty, like difficulty in transosseous suturing, poor tendon quality, knot slippage, and failure of primary repair [5,7]. In our case, we treated the distal patellar tendon rupture by combining repair with a suture anchor and augmentation with a Gracilis tendon.

## Case presentation

A 58-year-old male was brought to our hospital with complaints of pain, swelling, and difficulty in moving the right knee. He gave a history of a road accident in which he fell from a motorcycle seven days ago. The patient did not have any history of trauma to the same knee, nor any history of systemic disease or corticosteroid use.

**Figure 1:**
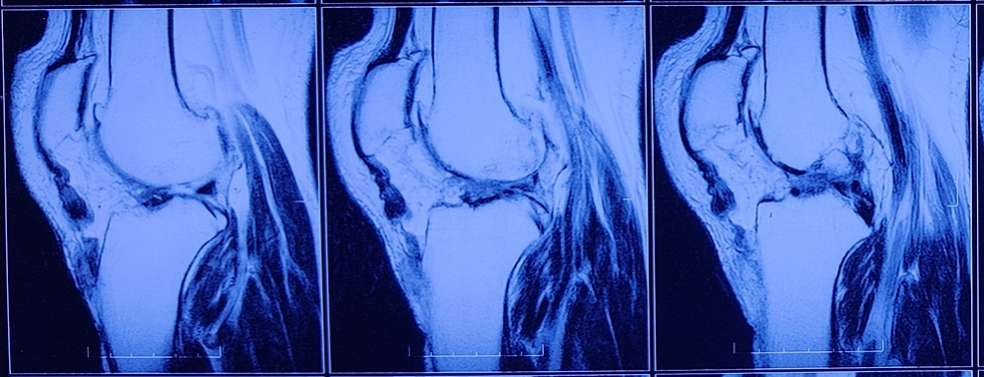
Sagittal magnetic resonance T2 imaging shows rupture of the patellar tendon at its attachment to the tibial tubercle

On examination, tense swelling and ecchymosis were noted around the right knee. There was a palpable defect over the patellar tendon. Tenderness was present over the tibial tubercle. Active extension was not possible. The patient was immobilized with a long knee brace and sent for MRI. An MRI T2 image revealed avulsion of the distal patellar tendon from the tibial tubercle insertion and a medial meniscus tear (Figure 1).

**Figure 2:**
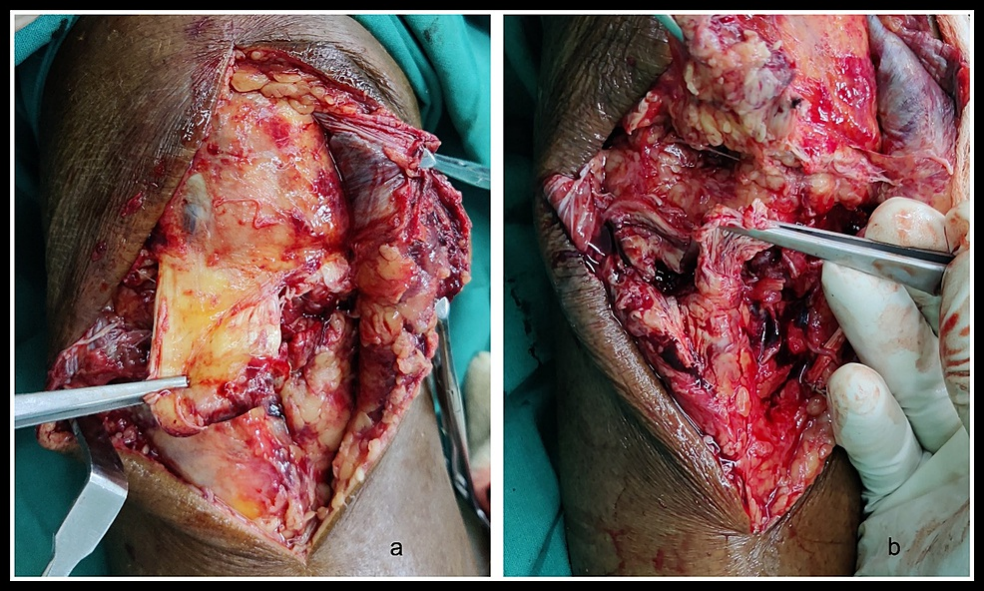
a) Complete rupture of the patellar tendon from the tibial tuberosity; b) Fibers remained attached to the tibial tuberosity medially

The patient had diffuse swelling around the injured knee so we took him for surgery five days later after reduction of the swelling. He first underwent diagnostic knee arthroscopy and arthroscopic partial medial meniscectomy. A midline skin incision was made over the right knee. Complete avulsion of the patellar tendon from the tibial tuberosity was present with a few fibers remaining attached to the tibial tuberosity medially (Figure 2).

**Figure 3:**
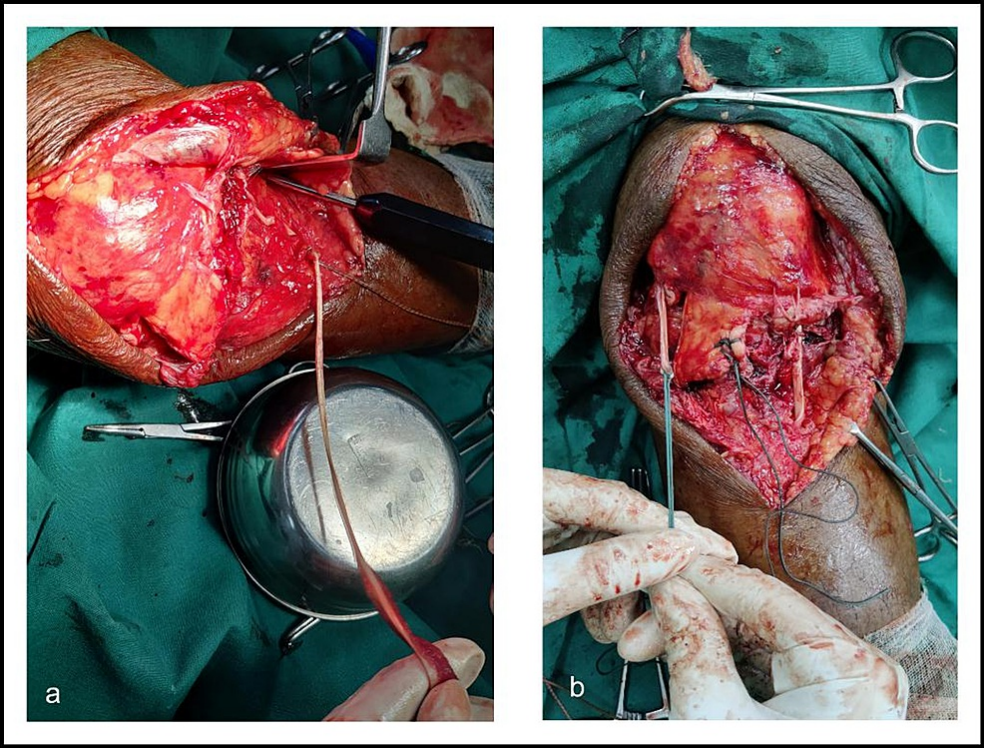
a) Gracilis tendon; b) Proximal end of the Gracilis tendon rerouted beneath the quadriceps tendon to the lateral side of the tibial tuberosity

The tendon attachment site on tuberosity was freshened up. The medial fibers that were attached to the tuberosity were fixed to the main tendon by Ethibond. The Gracilis tendon was harvested and the proximal end of the tendon was prepared and sutured with Ethibond. Distal tibial insertion of the Gracilis was kept intact. The proximal end of the tendon was rerouted beneath the quadriceps tendon to the lateral side of the tibial tuberosity (Figure 3).

**Figure 4:**
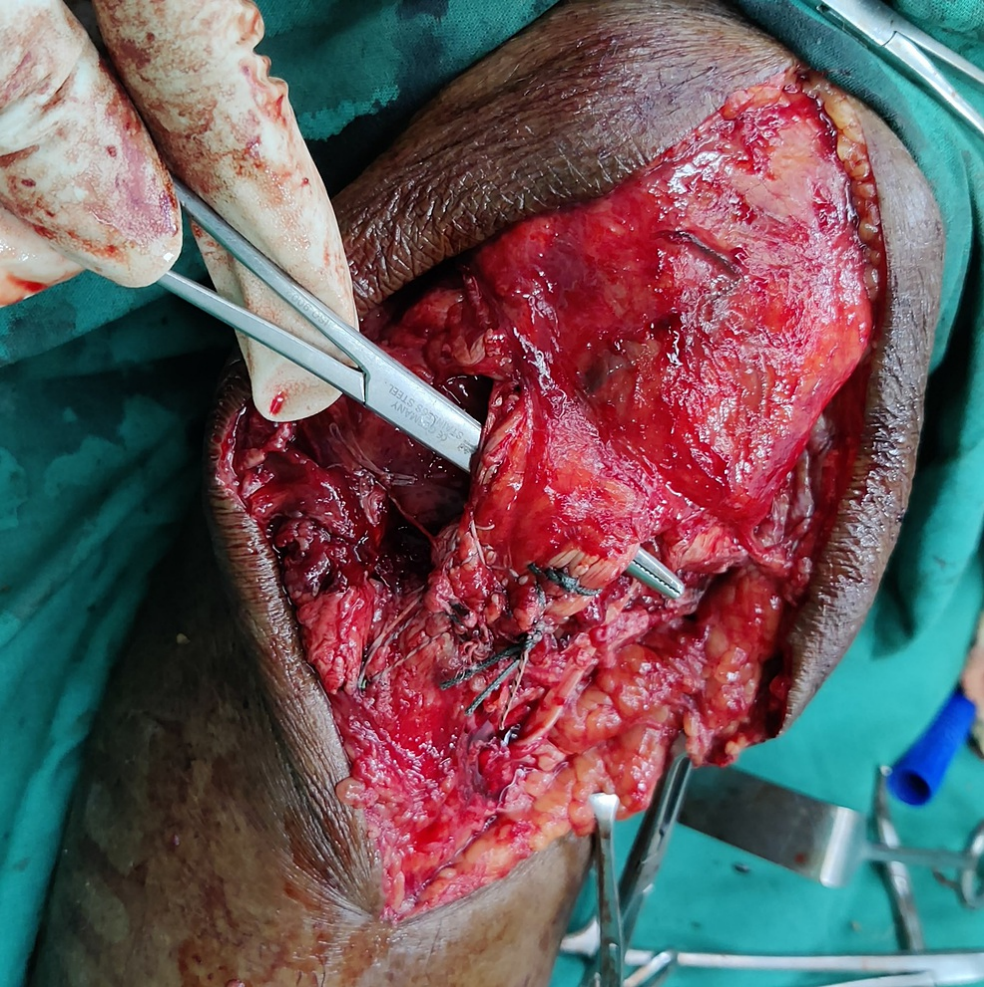
Final fixation of the patellar tendon

A double-loaded suture anchor with a needle was inserted laterally to the tibial tuberosity. The patella tendon was sutured to the tuberosity by suture threads. After primary repair of the patella tendon, the Gracilis tendon was sutured to the patella tendon and tuberosity (Figure 4).

Closure was done layer by layer. Postoperatively, a long knee brace was applied for the first three weeks. He was allowed to weight-bear as tolerated after three weeks with a hinged knee brace. Knee ROM and strengthening exercises were started after six weeks. After three months, the knee ROM reached up to 90 degrees, and there was no extension lag on straight leg raise. The patient resumed his daily routine as a farmer after six months with terminal restriction of knee flexion.

## Discussion

The incidence of patellar tendon ruptures from the tibial tuberosity is very uncommon [3-4]. A traumatic distal patellar tendon rupture in an adult is common in the third and fourth decades of life [1,3,5]. There are several systemic predisposing factors, such as chronic kidney insufficiency, diabetes, rheumatoid arthritis, fluoroquinolone treatment, hyperparathyroidism, corticosteroid injection [3,9-13], and local factors such as Osgood-Schlatter disease [4,7] and chronic tendinosis (jumper's knee) [14], for patellar tendon ruptures.

In our case, an injury occurred due to a road traffic accident, and there were no predisposing factors. Capogna et al. reported cases of distal patella tendon ruptures due to high-energy trauma in their case series [14]. There was a history of Osgood-Schlatter disease in patellar tendon avulsion from the tibial tubercle in the case report by Lang S D et al. [7] and in the case of the partial rupture distal patella tendon described by Cooper ME et al. [4]. In the case report by Chloros GD et al. [3], the patient directly fell anteriorly on a knee and sustained injury. Di Giacomo LM et al. reported an atraumatic rupture of the patellar tendon at the tibial tuberosity in a 52-year-old patient [9].

There are several surgical techniques available for the rupture of the patella tendon at the tibial tuberosity. In our case, our patient was 58 years old and the injury was also 13 days old. So we decided to go with a combination of primary repair of the tendon and Gracilis augmentation. We used one metal suture anchor with needle threads so that we could take sutures directly with the anchor threads. Di Giacomo et al. managed a case of atraumatic distal patella tendon rupture with primary repair only using fiber wire [9]. A case of patella tendon rupture from the tibial tuberosity with a history of Osgood-Schlatter disease was treated with primary repair and allograft augmentation by Lang S D et al. [7]. Avulsion of the patellar tendon from the tibial tubercle in a 52-year-old female treated with primary repair using a suture anchor was reported by Chloros GD et al [3].

Chloros GD et al. started physical therapy after six weeks of surgery, and the patient achieved full ROM after two and half months [3]. Lang SD et al. immediately started knee flexion up to 90 degrees till six weeks to avoid stiffness. Cooper et al. achieved sufficient strength and ROM after four weeks in a patient with a partial distal patellar tendon rupture treated by primary repair [4]. We started knee ROM exercises after six weeks because our patient is from a very remote and rural place where no physician or physiotherapist is available. After six months, the patient had a near-complete ROM with terminal restriction. The recovery of the patient was delayed due to irregular follow-up by the patient and the unavailability of trained personnel in the vicinity of the patient.

## Conclusions

Patellar tendon ruptures from the tibial tuberosity are infrequent. Various repair techniques have been described in the literature. There are no gold standard criteria for the repair technique due to the lack of data and the uncommon nature of injury. The goal of management is early mobilization with a strong repair. Hamstring augmentation with a primary repair is the safest and a good surgical option in elderly patients with patellar tendon ruptures from the tibial tuberosity.

## References

[1] Matava MJ (1996). Patellar tendon ruptures. J Am Acad Orthop Surg.

[2] Ramseier LE, Werner CM, Heinzelmann M (2006). Quadriceps and patellar tendon rupture. Injury.

[3] Chloros GD, Razavi A, Cheatham SA (2014). Complete avulsion of the patellar tendon from the tibial tubercle in an adult without predisposing factors. J Orthop Sci.

[4] Cooper ME, Selesnick FH (2000). Partial rupture of the distal insertion of the patellar tendon. A report of two cases in professional athletes. Am J Sports Med.

[5] Rothfeld A, Pawlak A, Liebler SA, Morris M, Paci JM (2018). Patellar tendon repair augmentation with a knotless suture anchor internal brace: a biomechanical cadaveric study. Am J Sports Med.

[6] Miyamoto S, Otsuka M, Hasue F (2017). Acute traumatic patellar tendon rupture at the tibial tuberosity attachment without avulsion fracture. Case Rep Orthop.

[7] Lang SD, Irons MR, Gilmer BB (2019). Repair of patellar tendon avulsion from the tibial tubercle: case report. J Orthop Case Rep.

[8] Rose MT, Caldwell PE 3rd, Vizzi S, Pearson SE (2021). Distal patellar tendon SpeedBridge repair. Arthrosc Tech.

[9] Di Giacomo LM, Khan MS, Bisaccia M, Rende R, Rinonapoli G, Caraffa A (2015). Surgical repair of an atraumatic avulsion of patellar tendon at the tibial tuberosity in an adult patient. Case Rep Orthop.

[10] Ramírez-Castillo HD, Carbajal-Contreras R, González-Morales DD (2010). Acute bilateral lesion of the patellar tendon associated to diabetes mellitus. Case report [Aricle in Spanish]. Acta Ortop Mex.

[11] Peiró A, Ferrandis R, Garcia L, Alcazar E (1975). Simultaneous and spontaneous bilateral rupture of the patellar tendon in rheumatoid arthritis. A case report. Acta Orthop Scand.

[12] van der Linden P, van Puijenbroek E, Feenstra J (2001). Tendon disorders attributed to fluoroquinolones: a study on 42 spontaneous reports in the period 1988 to 1998. Arthritis Care Res (Hoboken).

[13] Chen CH, Niu CC, Yang WE, Chen WJ, Shih CH (1999). Spontaneous bilateral patellar tendon rupture in primary hyperparathyroidism. Orthopedics.

[14] Capogna B, Strauss E, Konda S, Dayan A, Alaia M (2017). Distal patellar tendon avulsion in association with high-energy knee trauma: a case series and review of the literature. Knee.

